# Supplementary material for: PRMT1-mediated methylation of MICU1 determines the UCP2/3 dependency of mitochondrial Ca2+ uptake in immortalized cells
Source: Nat Commun. 2016 Sep 19;7:12897. doi: 10.1038/ncomms12897 (PMC5031806; doi:10.1038/ncomms12897)
Supplement: Supplementary Information — Supplementary Figures 1-23 and Supplementary Tables 1-3 [file ncomms12897-s1.pdf]

Supplementary Figure 1

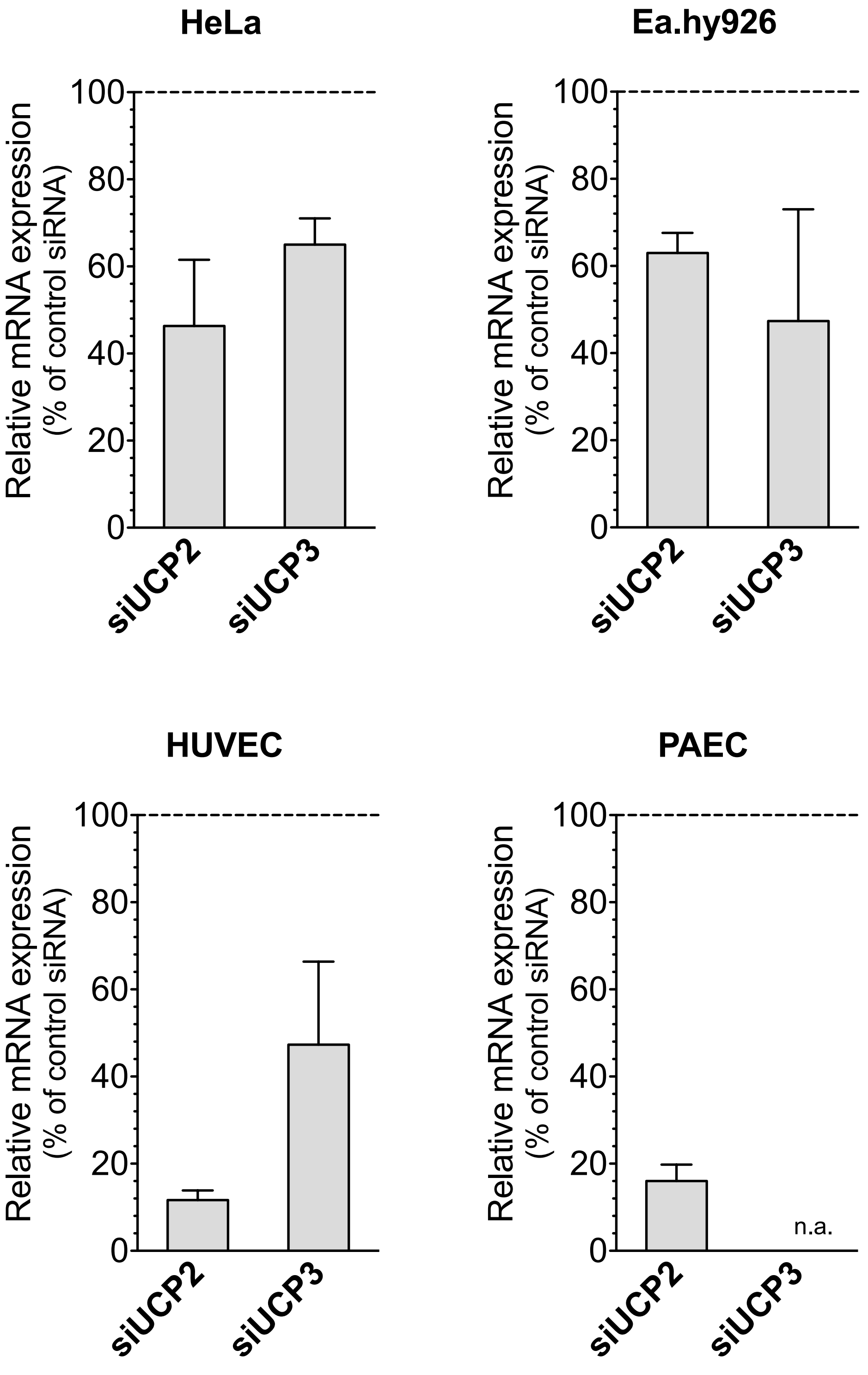

Relative mRNA expression levels. Bar charts show an average of relative mRNA expression levels (mean ± SEM) of HeLa, Ea.hy926, HUVEC and PAEC cells treated with control siRNA (*n=3*), siRNA against UCP2 (*n=3*) or UCP3 (*n=3*). UCP3 was undetectable in PAEC cells.

Supplementary Figure 2

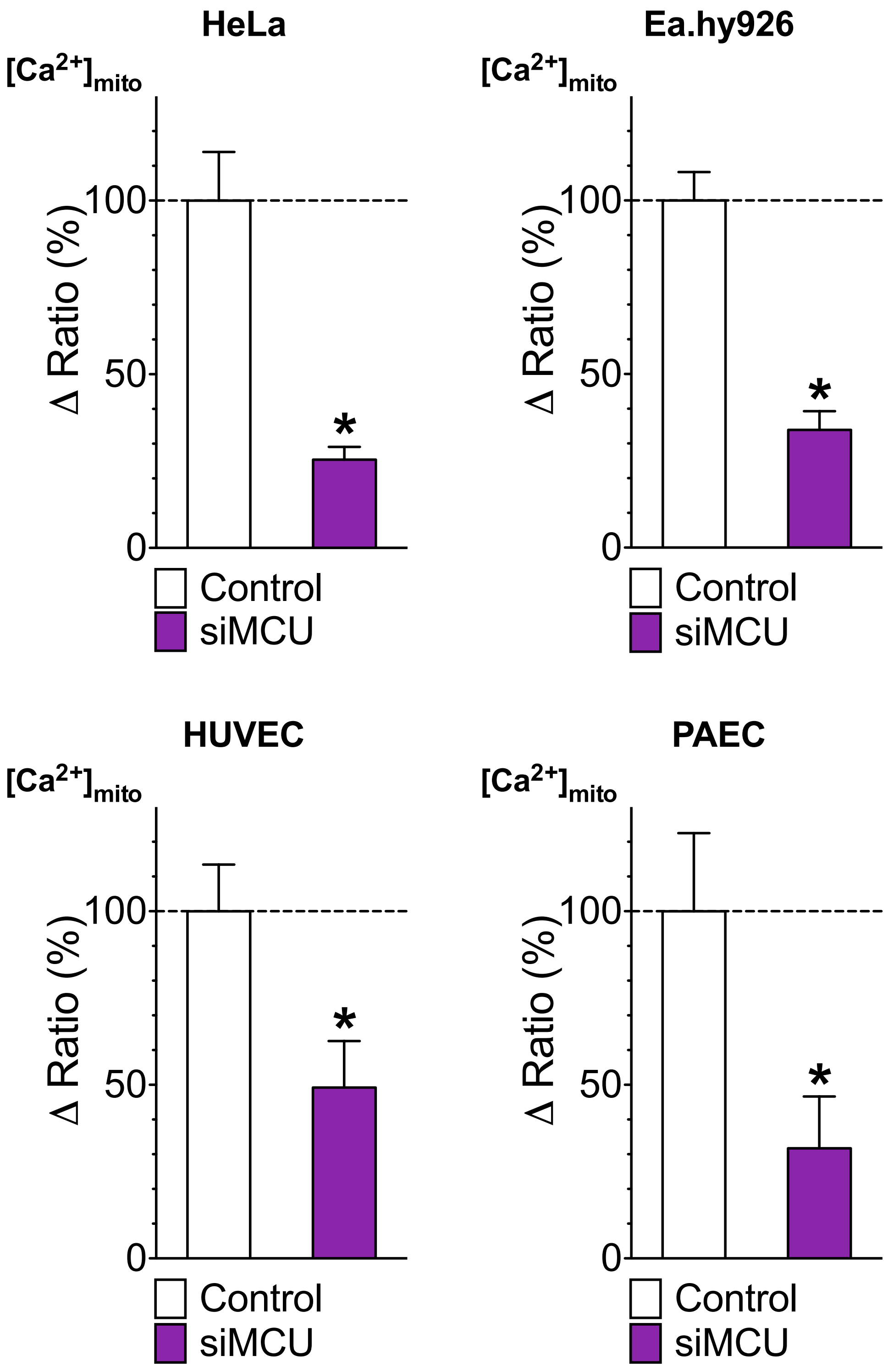

Mitochondrial Ca^{2+} uptake. Bars represent an average of maximal Δ ratio values of cells treated with siMCU upon cell stimulation with histamine in HeLa and Ea.hy926 or with ATP in HUVEC and PAEC cells. Data was calculated as percentage of maximal Δ ratio values of the corresponding control (white columns; Control: *HeLa: n=32/15, Ea.hy926: n= 16/8, HUVEC: n=19/5, PAEC: n=18/11*; violet columns; siMCU: *HeLa: 37/15, Ea.hy926: 19/7, HUVEC: 24/6, PAEC: 17/10*).

Supplementary Figure 3

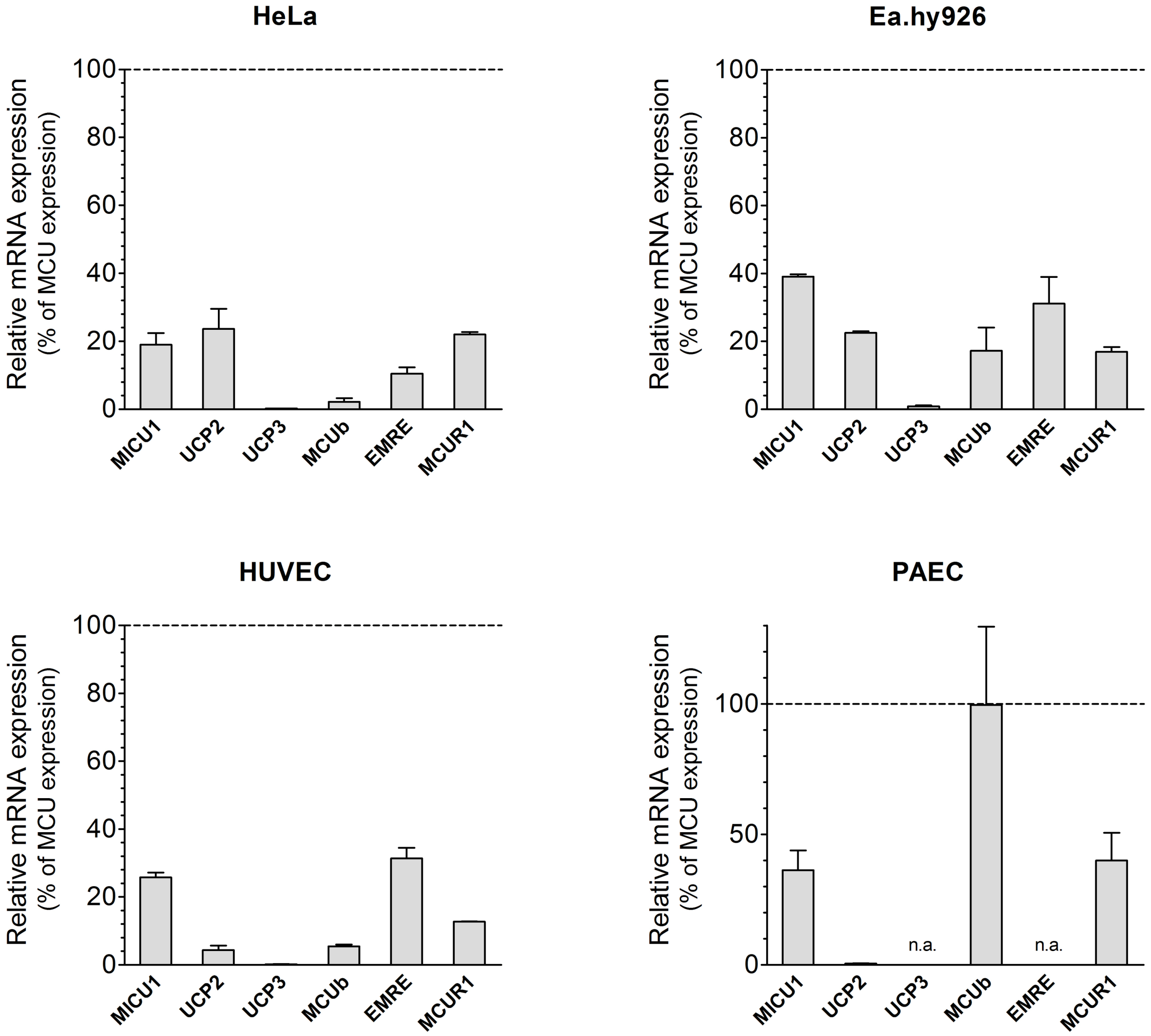

Relative mRNA expression level. The mRNA expression of known constituents of the MCU complex including MICU1, MCUb, EMRE and MCUR1, and UCP2 and UCP3 in HeLa, Ea.hy926, HUVEC and PAEC cells, calculated as percentage of MCU mRNA expression level (*n=3*). Bar charts indicate mean ± SEM.

Supplementary Figure 4

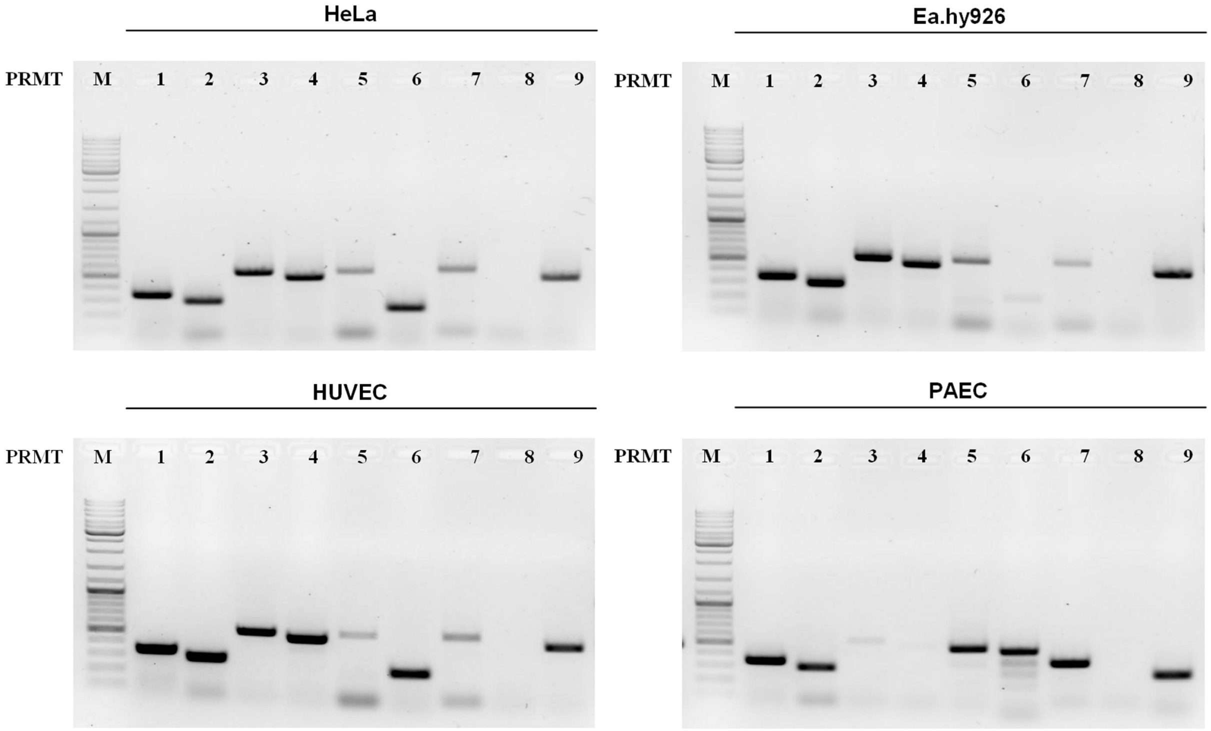

Detection PCR of PRMT isoforms. Representative gels show detection of mRNA levels of PRMT isoforms expressed in HeLa, Ea.hy926, HUVEC and PAEC cells via PCR using gene-specific primers. The PCR products were detected on 1.5% agarose gel using a 100 – 10.000-bp DNA ladder.

Supplementary Figure 5

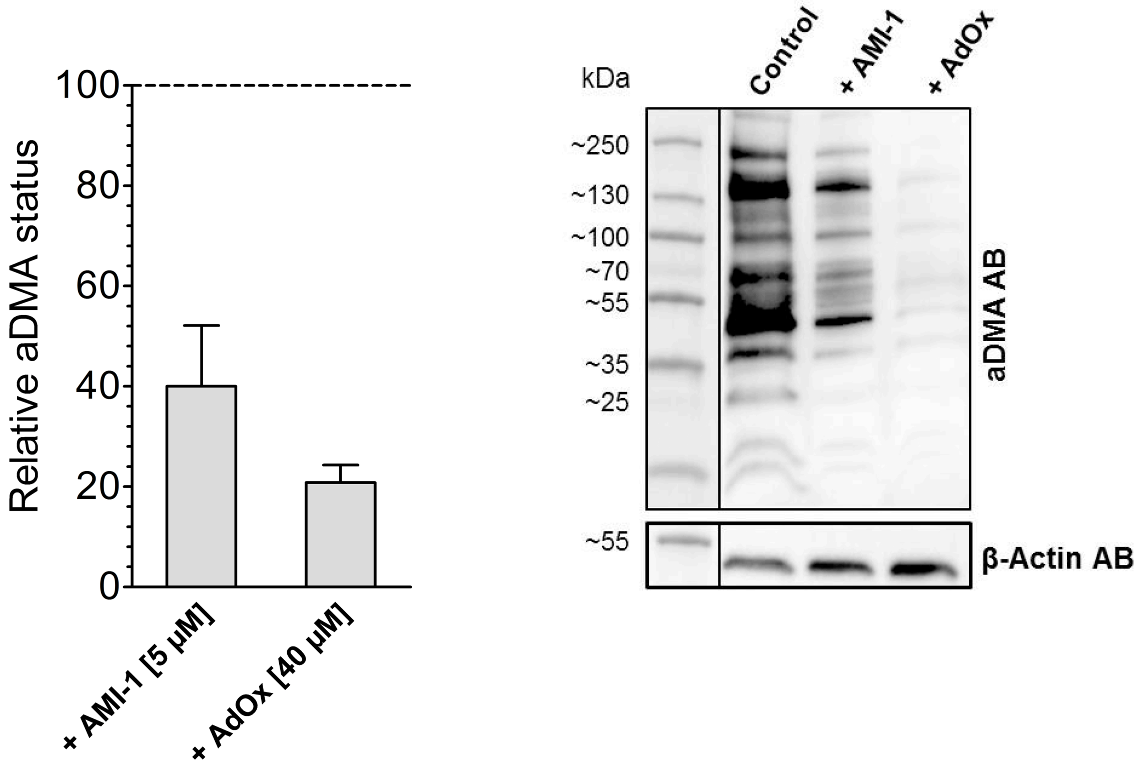

Left panel: **Percentage of asymmetric arginine dimethylation** in HeLa after treatment with 5 µM AMI-1 (*n=5*) or 40 µM AdOx (*n=7*) normalized to aDMA state of untreated HeLa. *Right panel:* Representative Western Blot revealing overall asymmetric dimethylation state by using aDMA antibody and housekeeping protein beta-actin level of untreated HeLa or HeLa after treatment with AMI-1 or AdOx.

Supplementary Figure 6

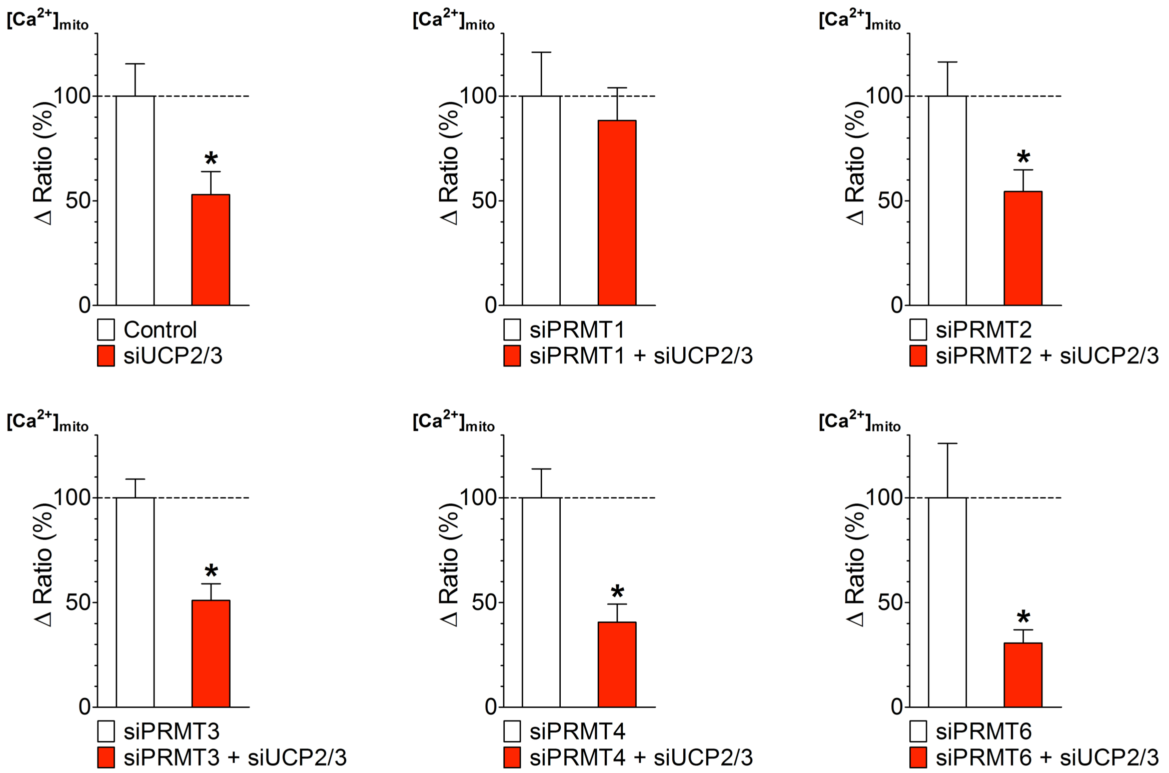

Mitochondrial Ca^{2+} uptake. Bars show an average of maximal Δ ratio values in response to 100 µM histamine in Ca^{2+}-free solution of 4mtD3cpv expressing control HeLa cells (*upper left panel*) or HeLa cells treated with siRNA against PRMT1 (*upper middle panel*), PRMT2 (*upper right panel*), PRMT3 (*lower left panel*), PRMT4 (*lower middle panel*) or PRMT6 (*lower right panel*), respectively, with or without a knockdown of UCP2/3. Data was calculated as percentage of maximal Δ ratio values of the corresponding control (white columns; *Control: n=48/6, siPRMT1: n=73/11, siPRMT2: n=86/13, siPRMT3: n=69/10, siPRMT4: n=38/11, siPRMT6: n=72/8;* red columns; *siUCP2/3: n=48/5, siPRMT1 + siUCP2/3: n=56/10, siPRMT2 + siUCP2/3: n=78/13, siPRMT3 + siUCP2/3: n=52/8, siPRMT4 + siUCP2/3: n=28/10, siPRMT6 + siUCP2/3: n=47/8*).

Supplementary Figure 7

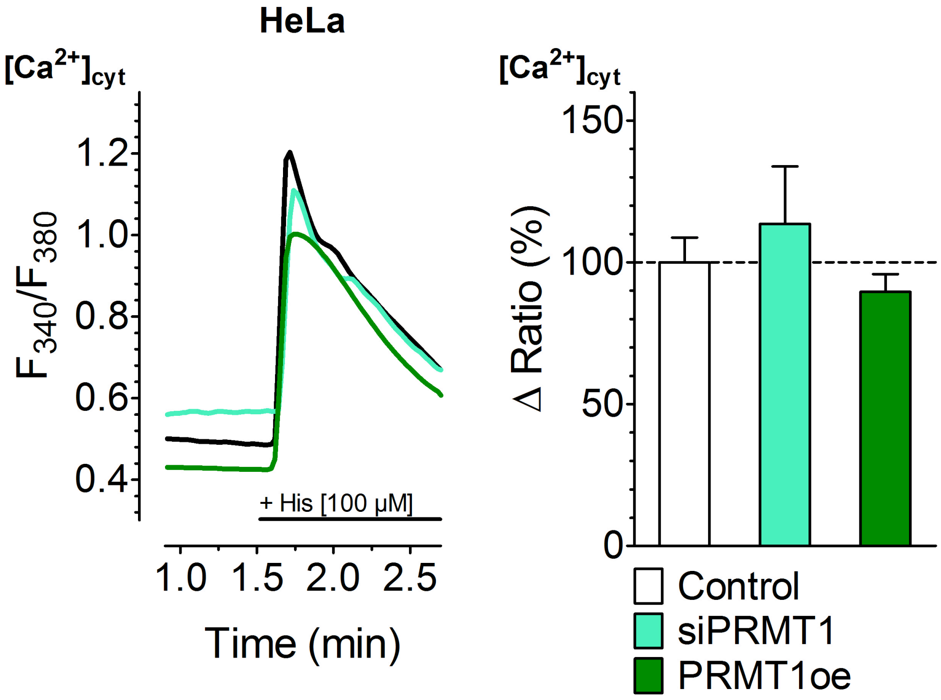

Cytosolic Ca^{2+} elevation. *Left panel:* Representative curves of cytCa^{2+} ratio signals over time in response to 100 µM histamine in Ca^{2+}-free solution of Fura-2/AM loaded HeLa cells treated with control siRNA (black curve) or with siRNA against PRMT1 (light green curve) as well as Hela cells overexpressing PRMT1 (dark green curve). *Right panel:* Bars represent an average of maximal Δ ratio signals of IP_3 generating agonist treatment of control HeLa cells (white column: *n=71/9*), cells depleted of PRMT1 (light green column: *n=92/9*) or overexpressing PRMT1 (dark green column: *n=66/3*).

Supplementary Figure 8

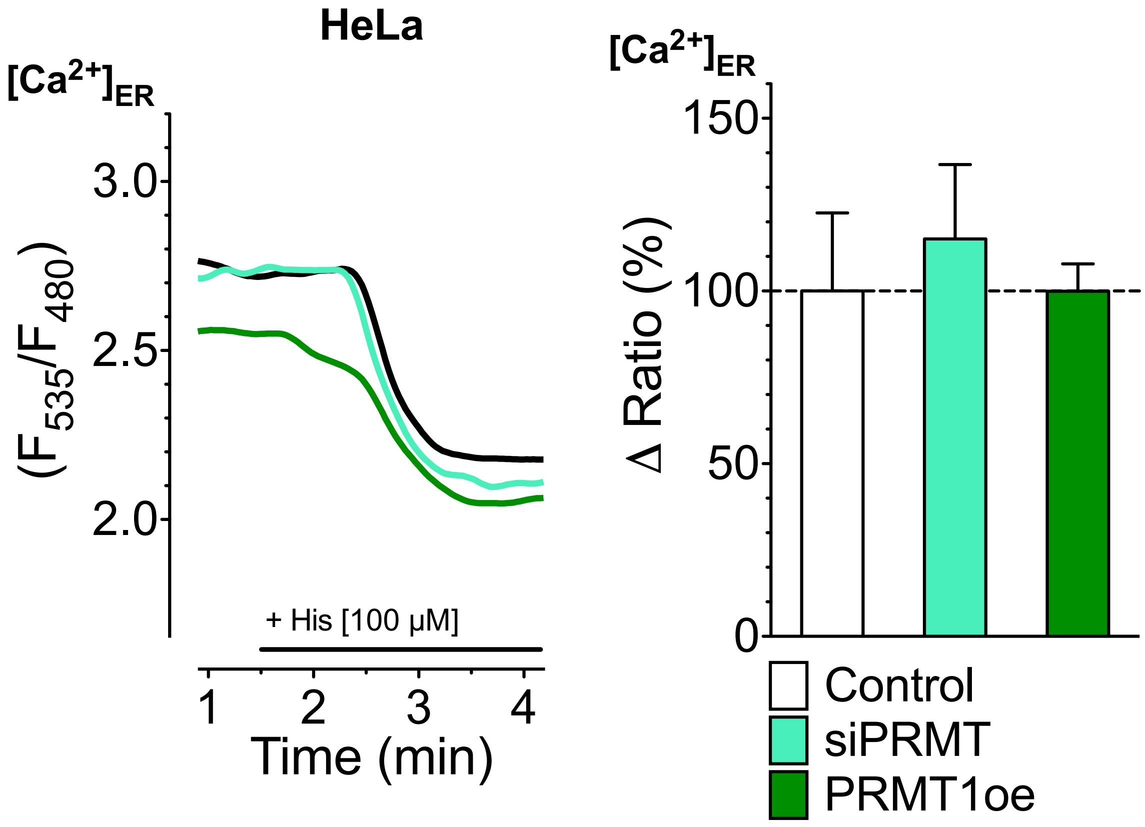

ER Ca^{2+} drop. *Left panel:* Representative curves of $ERCa^{2+}$ ratio signals over time, measured by D1ER in HeLa cells upon stimulation with 100 µM histamine, of control HeLa cells (black curve), with either siRNA against PRMT1 (light green curve) or overexpression of PRMT1 (dark green curve). *Right panel:*Bars represent an average of maximal Δ ratio signals of IP_3 generating agonist treatment of control HeLa cells (white column: *n=25/7*), with either siRNA against PRMT1 (light green column: *n=26/8*) or overexpression of PRMT1 (dark green column: *n=12/4*).

Supplementary Figure 9

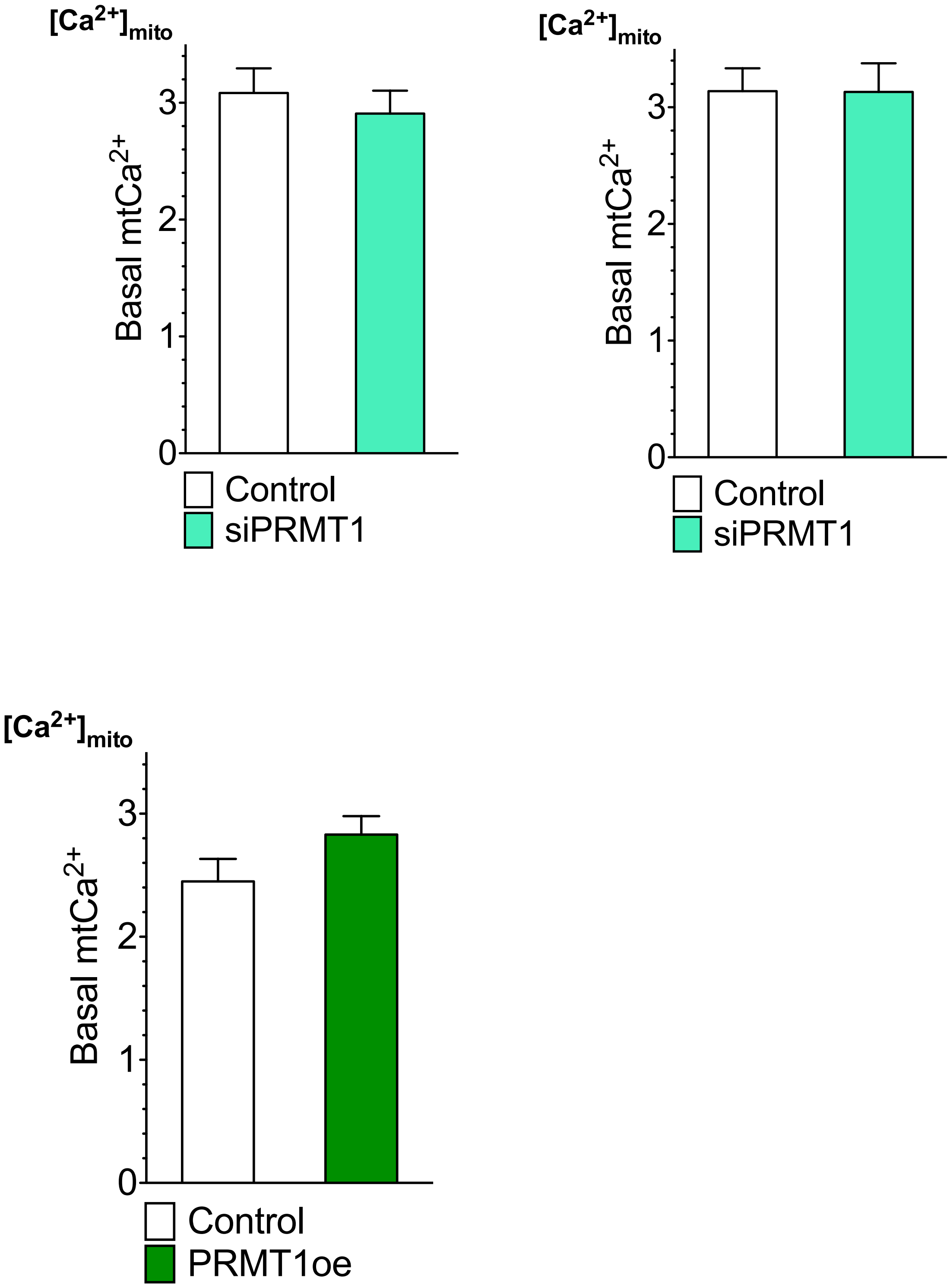

Basal Ca^{2+} levels of mitochondria in Ea.hy926 **(upper left panel)** and HeLa **(upper right panel)** cells with (light green columns; *Ea.hy926: n=31/12, HeLa: n=26/9*) or without knockdown of PRMT1 (white columns; *Ea.hy926: n=29/13, HeLa: n=35/13*). Bar charts indicate mean $\pm$ SEM. **(lower panel)** Basal mtCa^{2+} levels in control HUVEC cells (white column: *n=37/15*) or cells with PRMT1 overexpression (dark green column: *n=30/12*). Bar charts indicate mean $\pm$ SEM.

Supplementary Figure 10

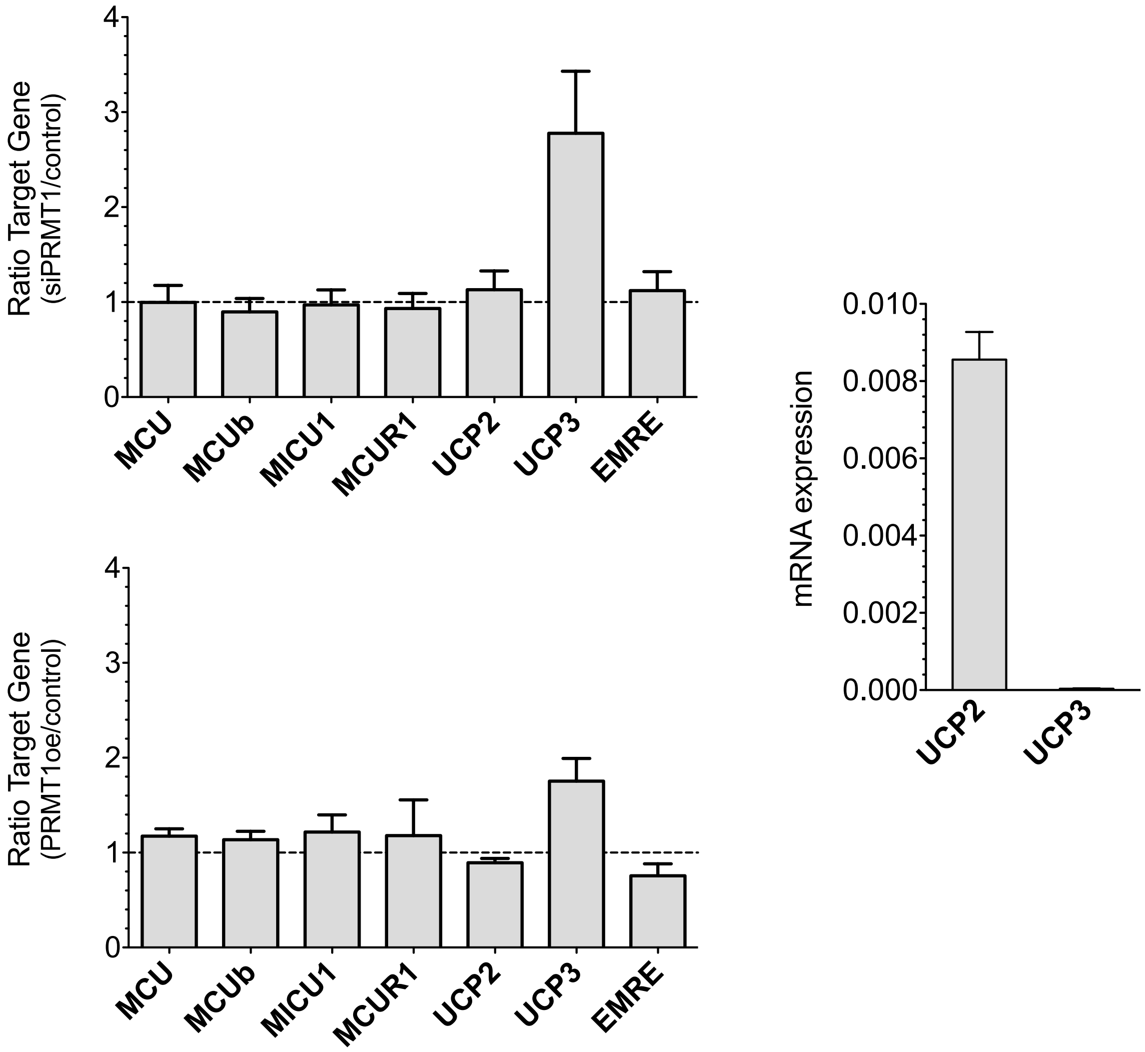

mRNA expression levels. *Left panels:* Ratio of mRNA expression of constituents of the MCU complex including MCU, MCUb, MICU1, MCUR1, UCP2, UCP3 and EMRE in cells with knockdown of PRMT1 *(upper panel)* or cells overexpressing PRMT1 (*lower panel*) in comparison to control cells. *Right panel:* Bars represent mRNA expression of UCP2 and UCP3 normalized to GAPDH as a house keeping gene *(n=3, each)*.

Supplementary Figure 11

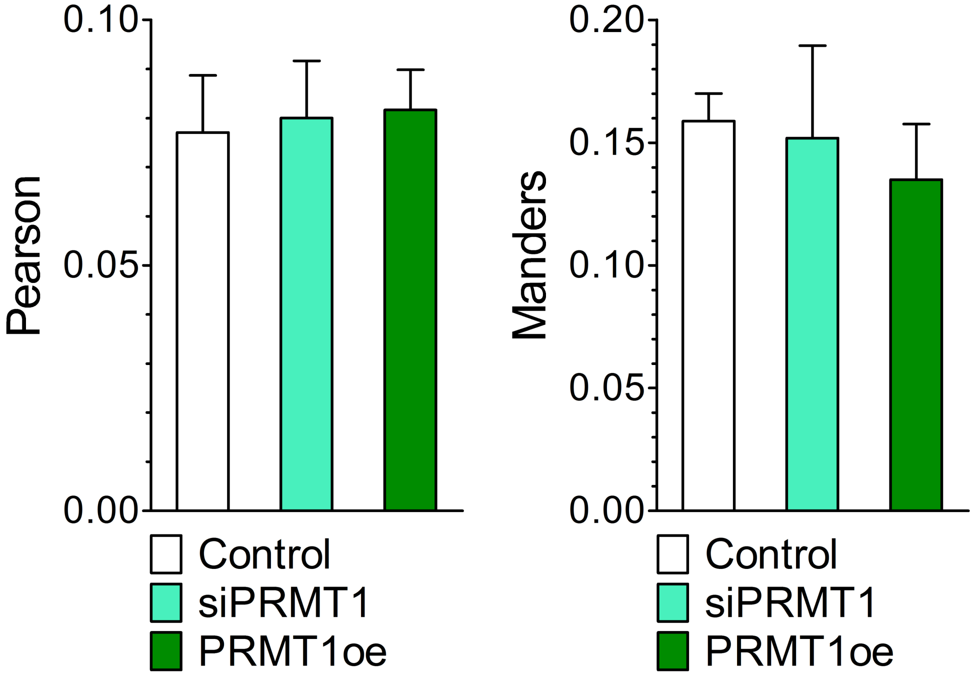

Co-localization levels of endoplasmic reticulum and mitochondria in HeLa cells after knockdown (light green column: *n=37/8*) or overexpression of PRMT1 (dark green column: *n=36/8*) compared to control (white column: *n=25/6*) represented by Pearson (*left panel*) and Costes corrected Manders coefficient (*right panel*).

Supplementary Figure 12

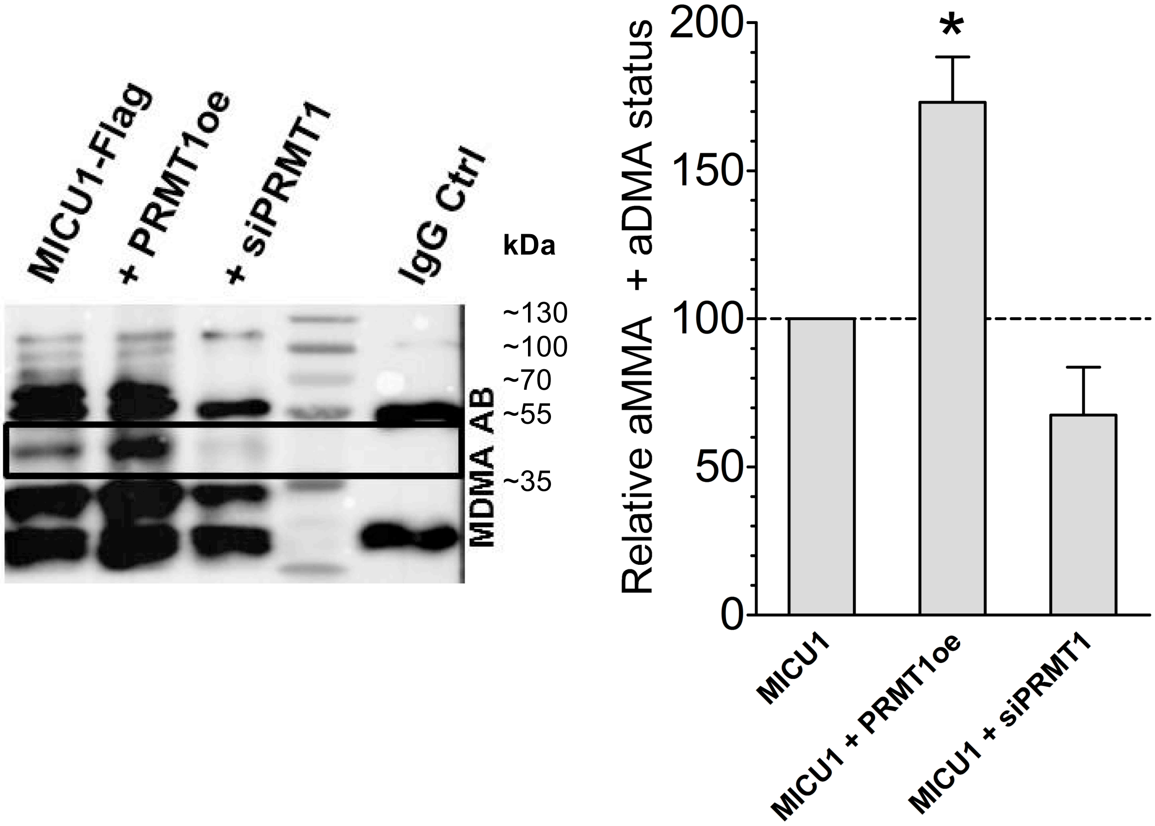

PRMT1-driven MICU1 methylation. *Left panel:* Representative Western Blots revealing arginine methylation of MICU1, after immunoprecipitation of flag-tagged proteins, by using an antibody against mono- and dimethyl arginines [MDMA], normalized to the intensities of detected MICU1 bands*. Right panel:* Bars represent the percentage of mono- and dimethyl arginine methylation of flag-tagged proteins of cells with knockdown or overexpression of PRMT1 normalized to mono- and dimethyl arginine methylation of flag-tagged proteins of control cells.

Supplementary Figure 13

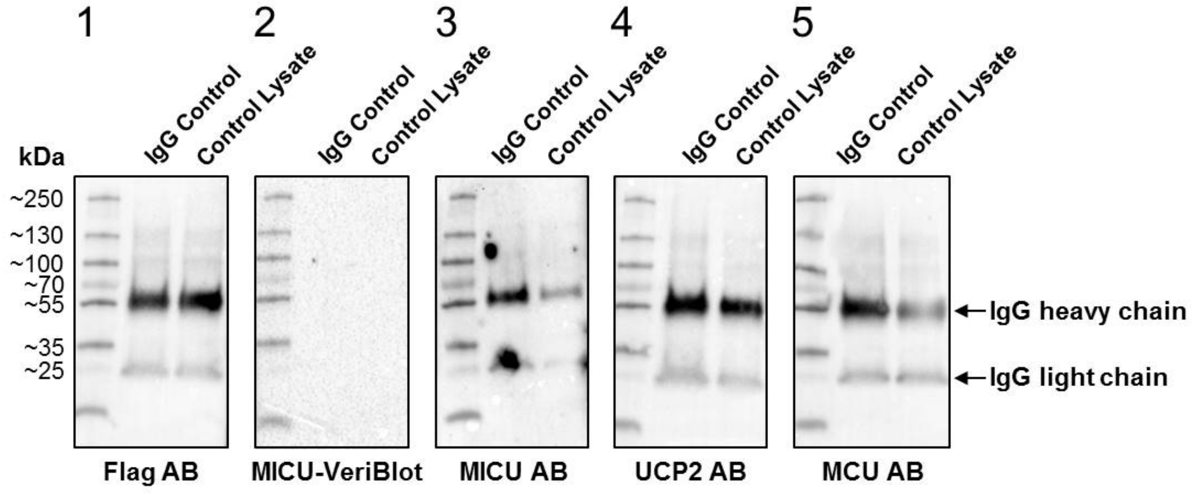

Western Blots showing control IgG and control lysates detected with antibodies against **(1)** Flag (r), **(2)** MICU1 and Veriblot as secondary antibody, **(3)** MICU1 (r), **(4)** UCP2 (m) and **(5)** MCU (r) to exclude unspecific bands due to impurities of immunoprecipitation, whereas (r) is rabbit and (m) is mouse secondary antibody. IgG heavy and light chains are marked with an arrow. VeriBlot for IP secondary antibodies only recognizes native (non-reduced) antibodies and therefore does not recognize IgG heavy and light chains.

Supplementary Figure 14

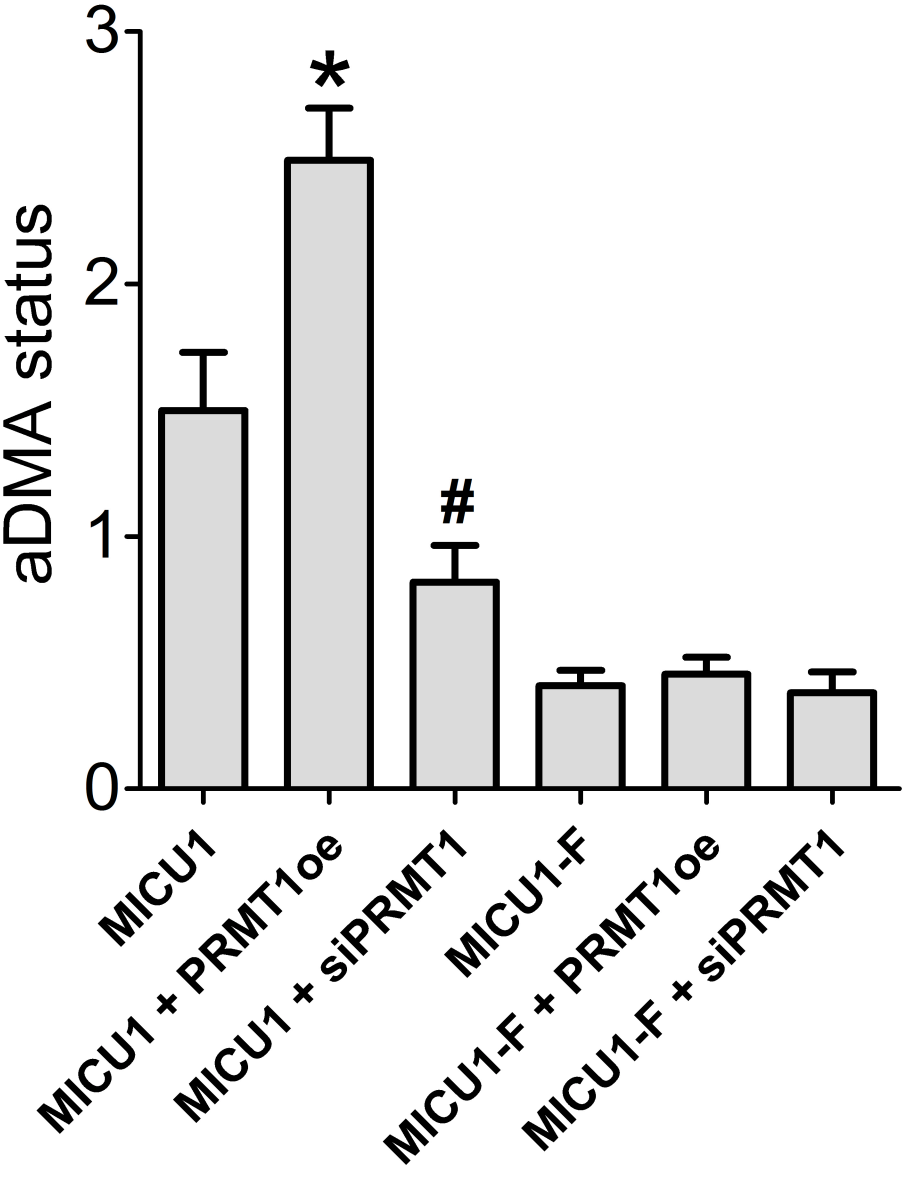

Bars represent the **aDMA status of flag-tagged MICU1-WT and MICU1-F** proteins of control cells or cells with either overexpression or knockdown of PRMT1 (*n=3*). After immunoprecipitation of flag-tagged proteins, aDMA status was determined by using an antibody against asymmetric dimethylated arginines aDMA and normalized to the intensities of detected bands using an anti-Flag antibody. The statistically significant different aDMA status between MICU1-WT and MICU1-WT+PRMT1 was marked with a star and the statistically significant difference between MICU1-WT and MICU1+siPRMT1 with a hash.

Supplementary Figure 15

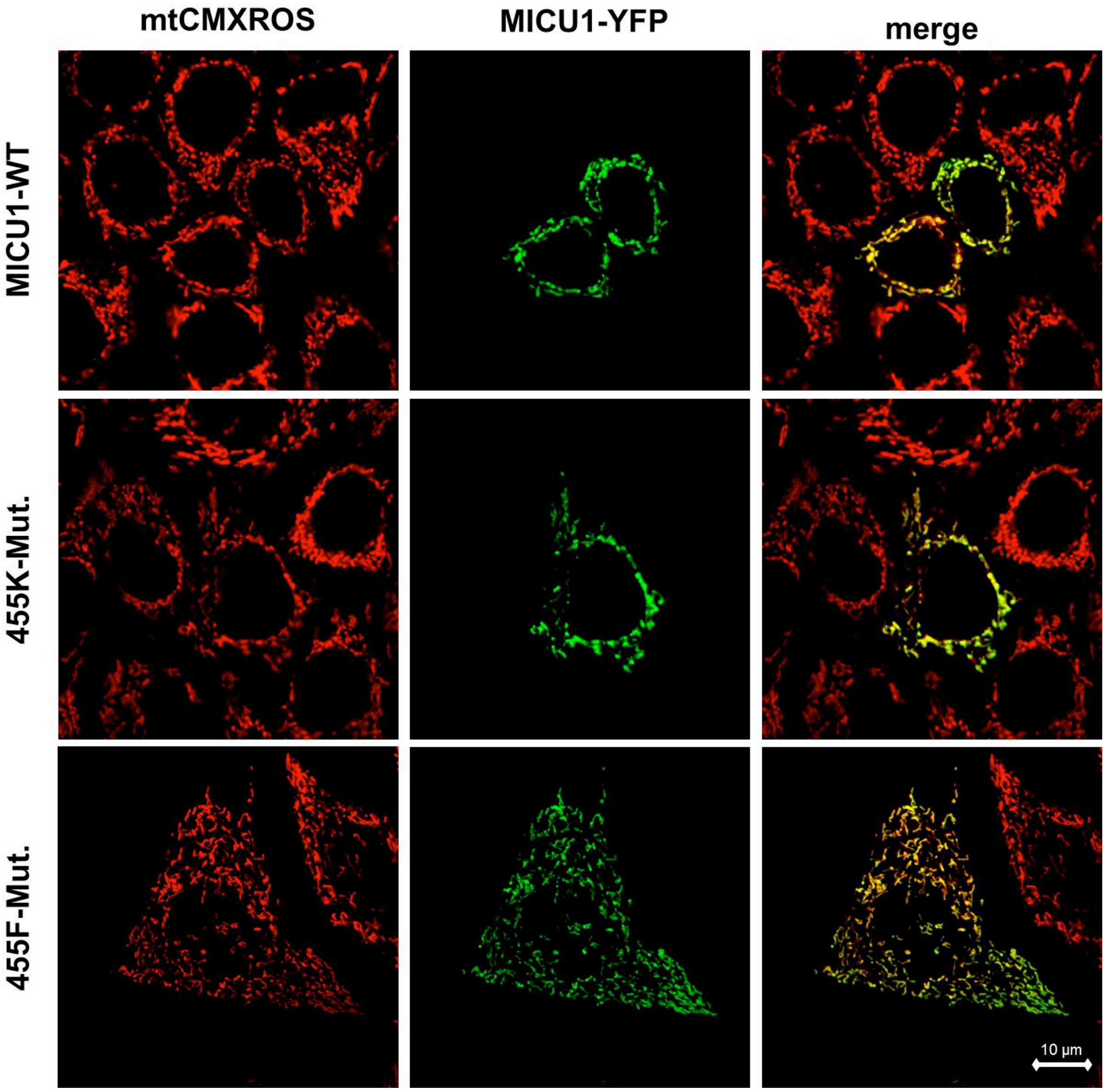

Representative confocal fluorescent images of HeLa cells expressing YFP-tagged MICU1-WT, 455F or 455K MICU1-mutants co-localizing with Mitotracker® Red CMXRos (mtCMXROS) stained mitochondria.

Supplementary Figure 16

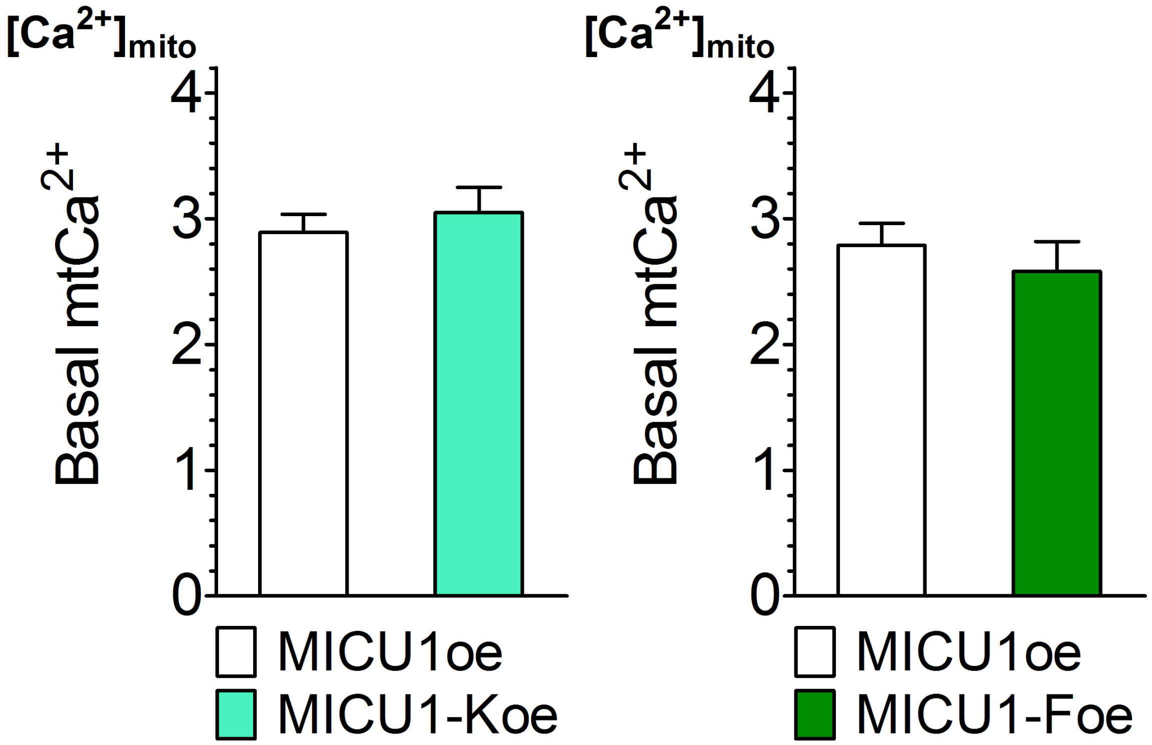

Basal mtCa^{2+} levels in HeLa cells (*left panel*) overexpressing MICU1-WT (white column: *n=68/16*) or 455K mutant (light green column: *n=39/16*) and HUVEC cells (*right panel*) overexpressing MICU1-WT (white column: *n=10/8*) or 455F mutant (dark green column: *n=13/6*).

Supplementary Figure 17

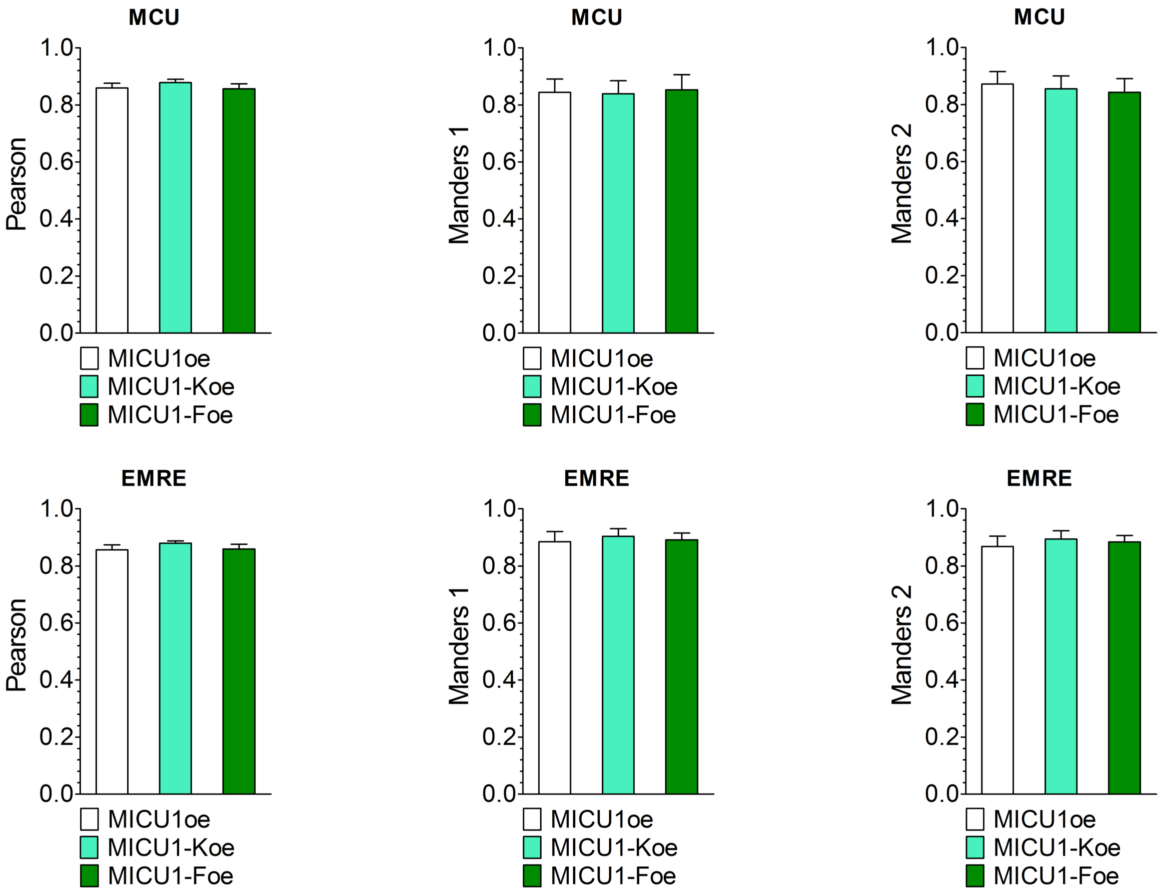

Co-localization of YFP-tagged MICU1-WT, MICU1-K or MICU1-F mutants with mCherry-tagged MCU or EMRE is unchanged. Pearson (*left panels*), Mander1 (*middle panels*) and Manders2 (*right panels*) co-localization coefficients of YFP-tagged MICU1-WT (white columns), MICU1-K (light green columns) or MICU1-F-mutants (dark green columns) with MCU-mCherry are shown in the upper three panels while co-localization coefficients with EMRE-mCherry are shown in the lower three panels (*n=30/9*). Bar charts indicate mean ± SEM.

Supplementary Figure 18

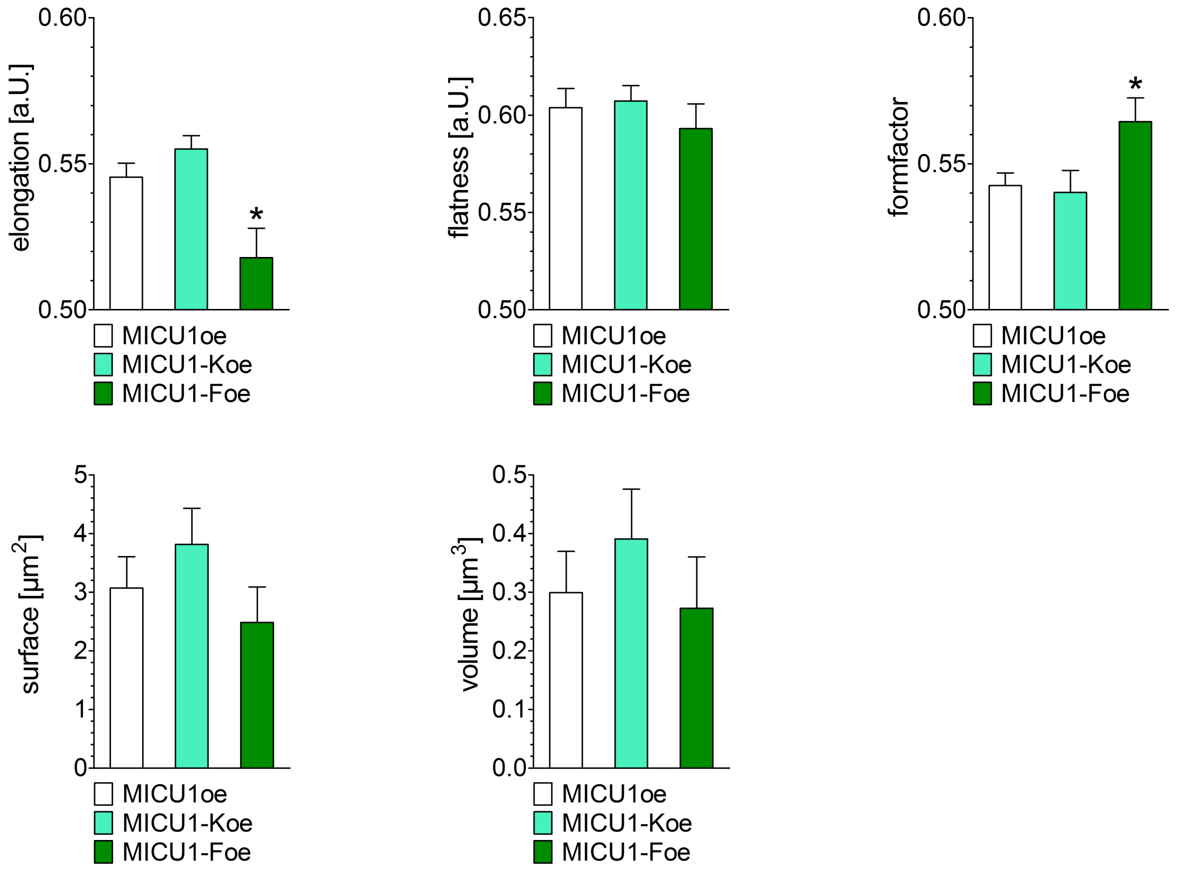

Overexpression of YFP-tagged MICU1-WT, MICU1-K or MICU1-F mutants affect mitochondrial morphology. Elongation (*left panel*), flatness (*middle panel*), formfactor (*right panel*), shown in the upper panels, as well as surface (*left panel*) and volume (*middle panel*), presented in lower panels, of MitoTracker® Red CMXRos labeled mitochondria are shown for cells overexpressing MICU1-WT (white columns), MICU1-K (light green columns) or MICU1-F-mutants (dark green columns). While surface, volume and flatness are unchanged, elongation is significantly decreased in cells with overexpression of YFP-tagged MICU1-F-mutant and mitochondrial branching is less complex (*n=30/9*). Bar charts indicate mean ± SEM.

Supplementary Figure 19

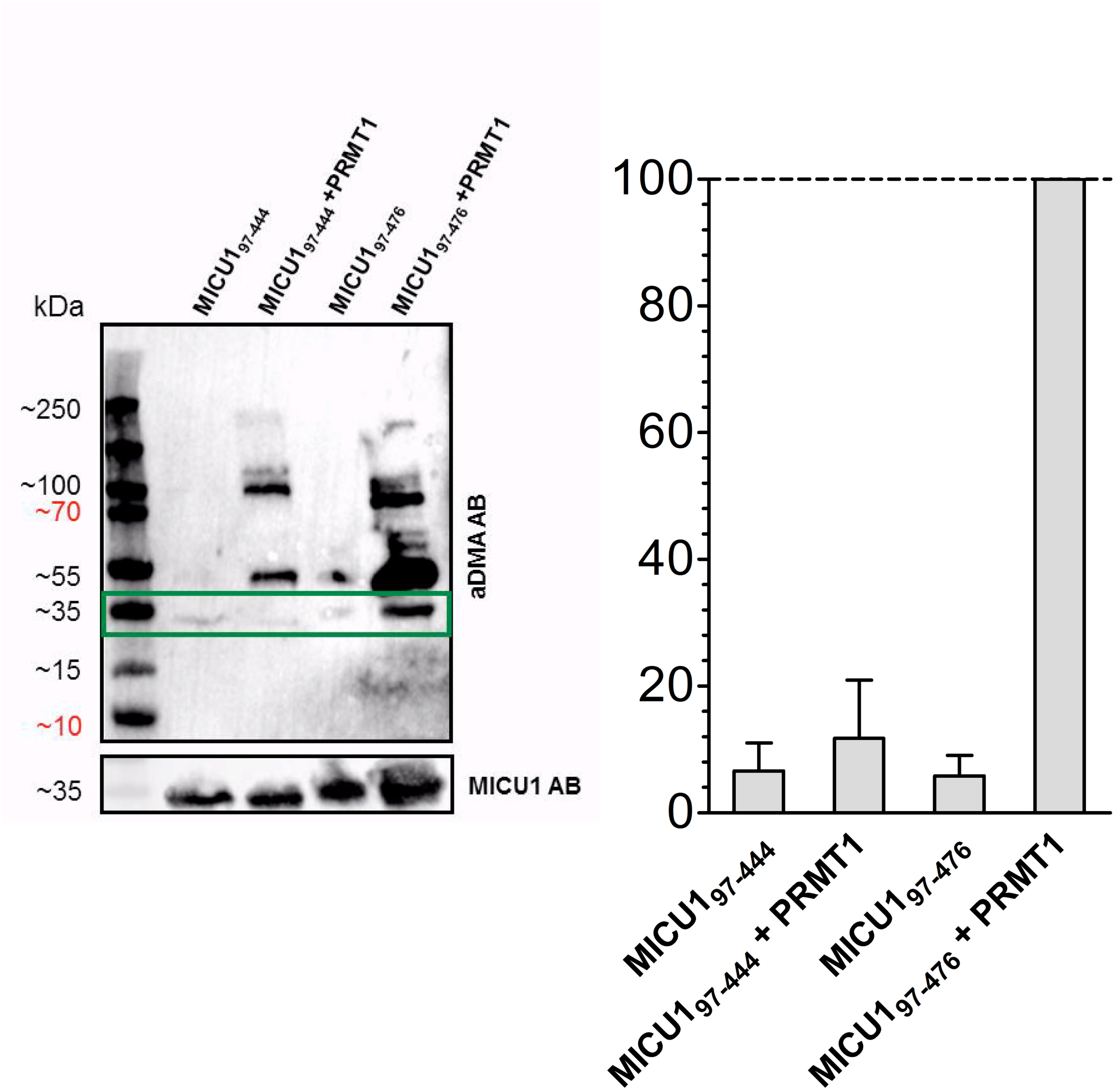

Left panel: Representative **Western Blot** shows purified recombinant MICU1 constructs MICU1$_{97\text{-}444}$ and MICU1$_{97\text{-}476}$ with or without prior in vitro methylation with recombinant PRMT1 using an antibody against asymmetric dimethyl arginine motif (aDMA). *Right panel:* Bars show percentage of aDMA of MICU1 constructs, normalized to methylated MICU1$_{97\text{-}476}$.

Supplementary Figure 20

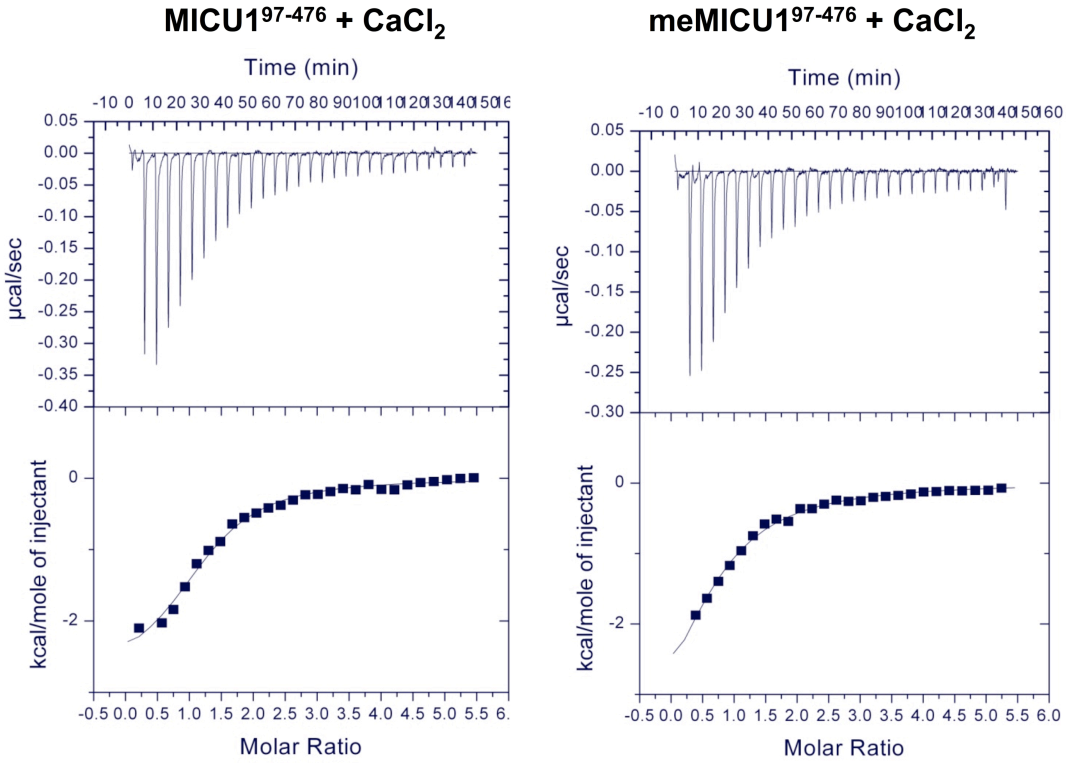

Isothermal titration calorimetry (ITC) titrations of MICU1$^{97\text{-}476}$ with $CaCl_2$. A 500 µM $CaCl_2$ solution was titrated into a 40 µM MICU1 solution and the heat release upon binding of $CaCl_2$ to MICU1 was detected. From the heat release, the characteristic thermodynamic parameters of interactions in solution including binding affinity can be determined. Experimental calorimetric data of the binding of $CaCl_2$ to MICU1$^{97\text{-}476}$ is shown. The arginine methylation status is indicated. The experiment was carried out at 25 °C. Dilution heats measured by titrating $CaCl_2$ into the corresponding buffer were in the range of the heat effects observed at the end of the titration (data not shown) and were subtracted for the analysis.

Supplementary Figure 21

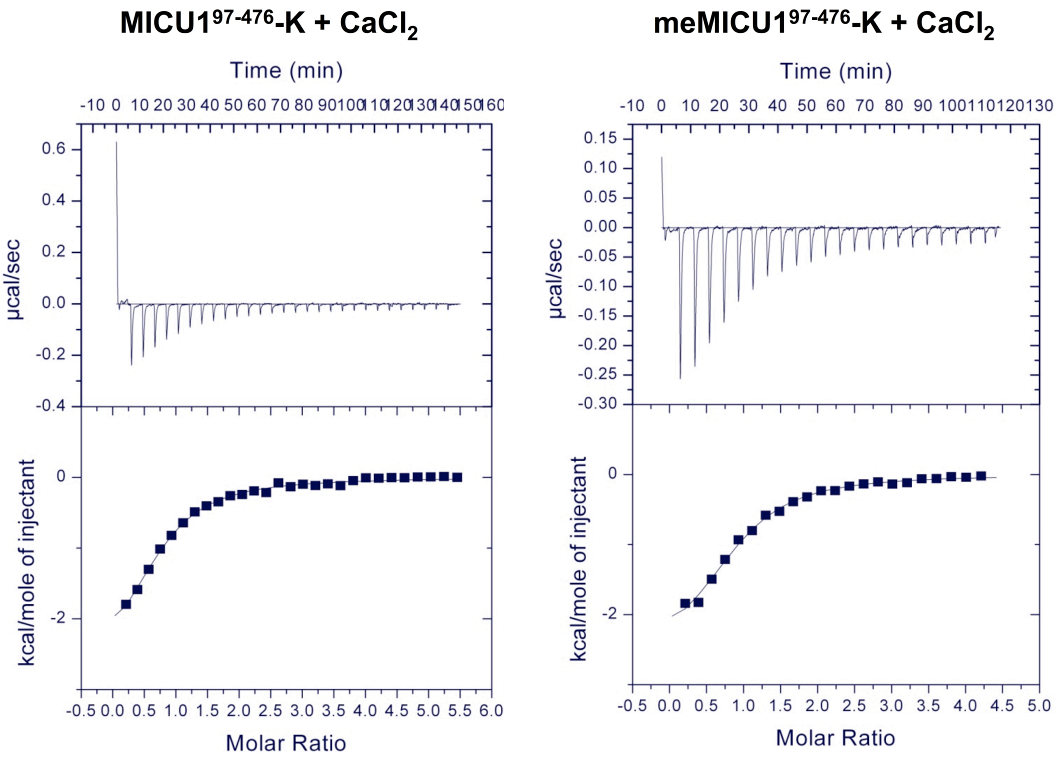

Isothermal titration calorimetry (ITC) titrations of MICU1$^{97\text{-}476}$-K with $CaCl_2$. A 500 µM $CaCl_2$ solution was titrated into a 40 µM MICU1$^{97\text{-}476}$-K solution and the heat release upon binding of $CaCl_2$ to MICU1$^{97\text{-}476}$-K was detected. From the heat release, the characteristic thermodynamic parameters of interactions in solution including binding affinity can be determined. Experimental calorimetric data of the binding of $CaCl_2$ to MICU1$^{97\text{-}476}$-K is shown. The arginine methylation status is indicated. The experiment was carried out at 25 °C. Dilution heats measured by titrating $CaCl_2$ into the corresponding buffer were in the range of the heat effects observed at the end of the titration (data not shown) and were subtracted for the analysis.

Supplementary Figure 22

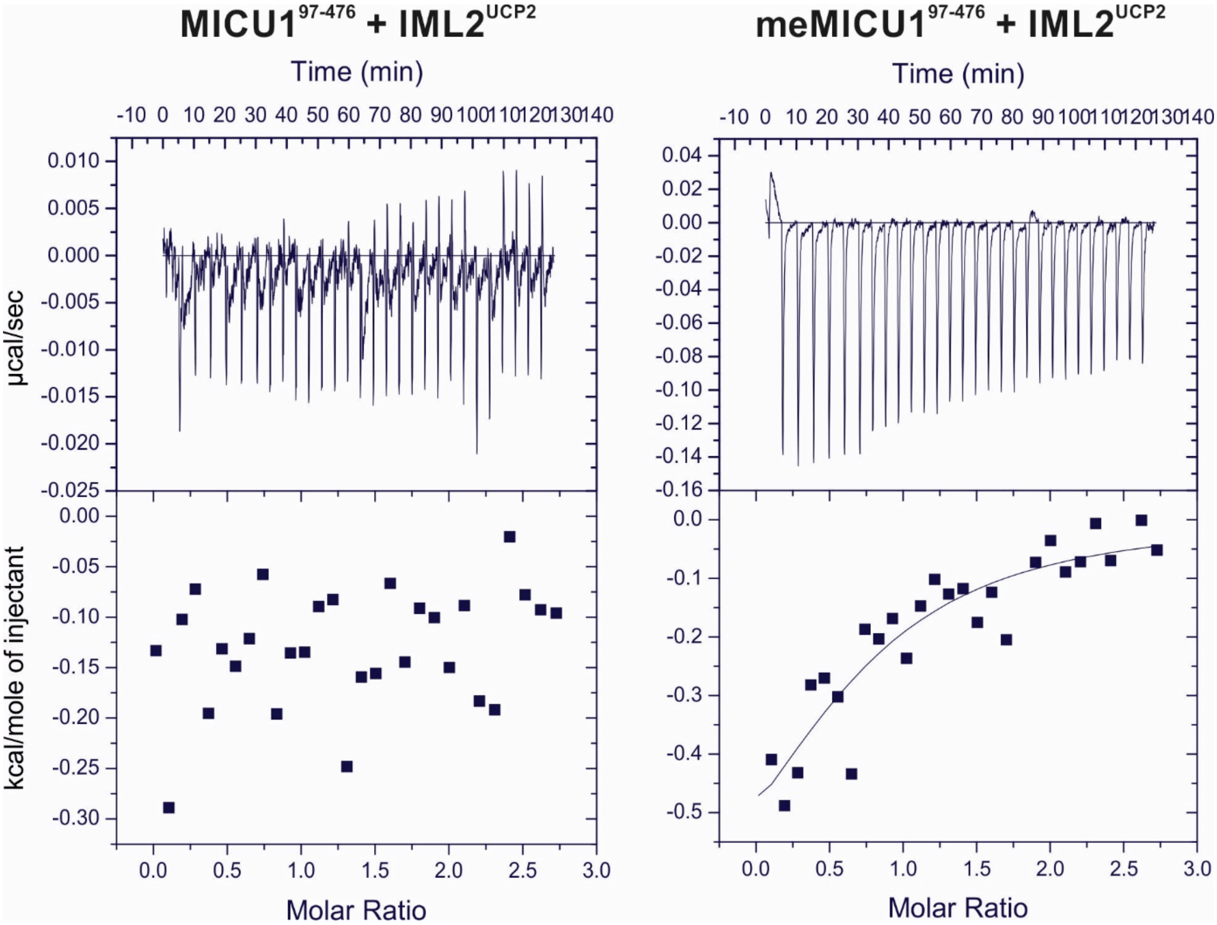

ITC titrations of MICU1$^{97-476}$ with IML2^{UCP2}. A 500 µM IML2^{UCP2} solution was titrated into a 40 µM MICU1 solution and the heat release upon binding of IML2^{UCP2} to MICU1 was detected. Experimental calorimetric data of the binding of IML2^{UCP2} to MICU1$^{97-476}$ is shown. The arginine methylation status is indicated. The experiment was carried out at 25 °C. Dilution heats measured by titrating IML2^{UCP2} into the corresponding buffer were in the range of the heat effects observed at the end of the titration (data not shown) and were subtracted for the analysis.

Supplementary Figure 23

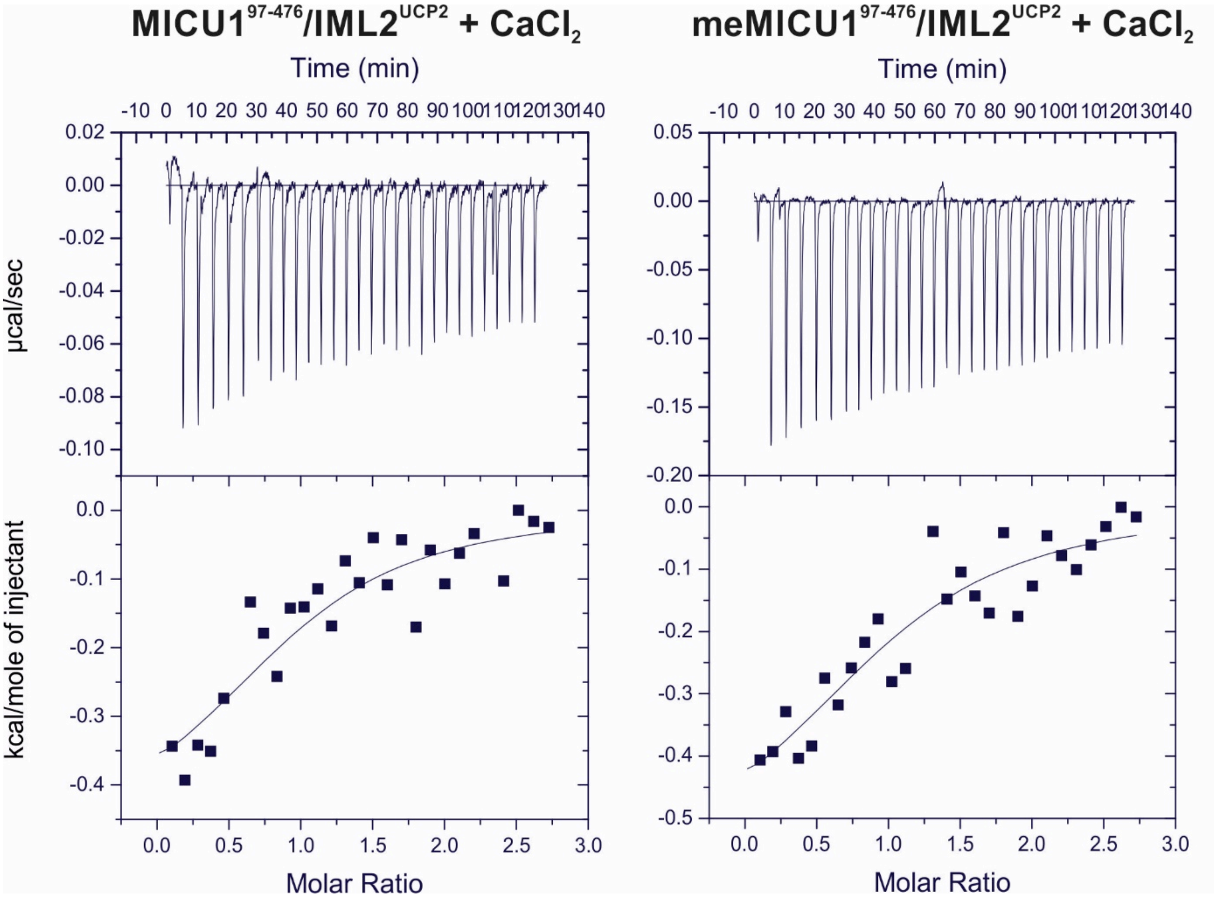

ITC titrations of MICU1$^{97-476}$/IML2^{UCP2} with $CaCl_2$. A 500 µM $CaCl_2$ solution was titrated into a 40 µM MICU1/100 µM IML2^{UCP2} solution and the heat release upon binding of CaCl2 to MICU1/IML2^{UCP2} was detected. Experimental calorimetric data of the binding of $CaCl_2$ to MICU1$^{97-476}$/IML2^{UCP2} is shown. The arginine methylation status is indicated. The experiment was carried out at 25 °C. Dilution heats measured by titrating $CaCl_2$ into the corresponding buffer were in the range of the heat effects observed at the end of the titration (data not shown) and were subtracted for the analysis.

Supplementary Table 1. Bioinformatic prediction of arginine methylation sites.

Protein name	Position of site	Flanking residues	SVM Probability[1]
**EMRE** (Q9H4I9)	52	SRSVIVT-**R**-SGAILPK	0.590069
**MICU1** (Q9BPX6)	31	SQPIQIR-**R**-RLMMVAF	0.573870
	32	QPIQIRR-**R**-LMMVAFL	0.629859
	54	STGLLWK-**R**-AHAESPP	0.688593
	117	KVMEYEN-**R**-IRAYSTP	0.529285
	119	MEYENRI-**R**-AYSTPDK	0.550184
	129	STPDKIF-**R**-YFATLKV	0.574960
	175	LDQYIIK-**R**-FDGKKIS	0.506645
	221	TVLSTPQ-**R**-NFEIAFK	0.527677
	261	TSMGMRH-**R**-DRPTTGN	0.597050
	263	MGMRHRD-**R**-PTTGNTL	0.500000
	440	FVSIMKQ-**R**-LMRGLEK	0.624701
	443	IMKQRLM-**R**-GLEKPKD	0.574625
	455	PKDMGFT-**R**-LMQAMWK	0.592892
**MCU** (Q8NE86)	16	LLLLLSS-**R**-GGGGGGA	0.682903
	54	HHRTVHQ-**R**-IASWQNL	0.535384
	89	GLPVISV-**R**-LPSRRER	0.615331
	231	ISRKAEK-**R**-TTLVLWG	0.562755
	297	VYPEARD-**R**-QYLLFFH	0.546431
**UCP2** (P55851)	60	ATASAQY-**R**-GVMGTIL	0.645166
	143	PTDVVKV-**R**-FQAQARA	0.566124
	149	VRFQAQA-**R**-AGGGRRY	0.718283
	267	MLQKEGP-**R**-AFYKGFM	0.500000
	279	GFMPSFL-**R**-LGSWNVV	0.628935
	296	VTYEQLK-**R**-ALMAACT	0.543824

[1] Predictions were carried out using the PMeS web tool (http://bioinfo.ncu.edu.cn/inquiries_PMeS.aspx) (Shi S-P et al. PLoS ONE 7(6): e38772, 2012). This tool identifies protein methylation sites based on an enhanced feature encoding scheme and a support vector machine (SVM; 1…high probability, 0…low probability). A window size ranging from -7 to +7 residues is employed to construct the prediction model.

Supplementary Table 2. Validation of predicted arginine methylation sites.

Protein name	Position	Conservation	Surface exposure[2]	Regulatory function
MICU1 (Q9BPX6)	31	conserved	exposed	close to transmembrane region
	32	conserved	exposed	close to transmembrane region
	54	conserved	exposed	unknown
	117	highly conserved	exposed	unknown
	119	highly conserved	buried	unknown
	129	highly conserved	exposed	unknown
	175	highly conserved	exposed	unknown
	221	highly conserved	exposed	close to oligomerization interface
	261	highly conserved	exposed	unknown
	263	conserved	exposed	unknown
	440	highly conserved	buried	unknown
	443	highly conserved	buried	unknown
	**455**	**highly conserved**	**exposed**	**oligomerization**

[2] Surface exposure was determined by visual inspection of the MICU1 crystal structure (PDB 4NSC).

Supplementary Table 3. List of primers used in this study

**Real-time PCR**	hEMRE	forward:	5'-TCGCTGGCTAGTATTGGCAC-3'
		reverse:	5'-GGAGAAGGCCGAAGGACATT-3'
	hMICU1	forward:	5'-CAGGTTCAGAGCATCATTCG-3'
		reverse:	5'-GAACACAAGCCAGACTTGAG-3'
	hMCU	forward:	5'-AGAGATAGGCTTGAGTGTGAAC-3'
		reverse:	5'-TTCCTGGCAGAATTTGGGAG-3'
	hMCUb	forward:	5'-TATAGTACCGTGGTGCCACCTGATG-3'
		reverse:	5'-TTGTAGGTCCTGAAGGAATGAACCA-3'
	hMCUR1	forward:	5'-GCAGGAGAGAGCTAAGCTTG-3'
		reverse:	5'-GGCATGAGTGTCGAAGTAGA-3'
	hPRMT1	forward:	5'-TGCTCAACACCGTGCTCTATGC-3'
		reverse:	5'-TCCTCGATGGCCGTCACATACA-3'
	hUCP2	forward:	5'-TCCTGAAAGCCAACCTCATG-3'
		reverse:	5'-GGCAGAGTTCATGTATCTCGTC-3'
	hUCP3	forward:	5'-AGAAAATACAGCGGGACTATGG-3'
		reverse:	5'-CTTGAGGATGTCGTAGGTCAC-3'
	sGAPDH	forward:	5'-TGGTGAAGGTCGGAGTGAAC-3'
		reverse:	5'-TGACTGTGCCGTGGAACTTG-3'
	sMCU	forward:	5'-GATAGACCTCCTTCTCCTTGACGAC-3'
		reverse:	5'-GTGTATAGCTGCTGGACCAGTGTCT-3'
	sMCUb	forward:	5'-TATAGTACCGTGGTGCCACCTGATG-3'
		reverse:	5'-TTGTAGGTCCTGAAGGAATGAACCA-3'
	sMCUR1	forward:	5'-GTCTGCCTTGGTCAAGATCACAGAG-3'
		reverse:	5'-TAACTGCTGAAGCGTGATCTCCTGC-3'
	sMICU1	forward:	5'-GGAGATGGAGAAGTAGACATGGAGG-3'
		reverse:	5'-CAAGCCAGACTTGAGGGTGTTACC-3'
	sUCP2	forward:	5'-CACTGTCGACGCCTACAAGACCATC-3'
		reverse:	5'-GTCATAGGTCACCAGCTCAGCACAG-3'
**Detection PCR**	hPRMT1	forward:	5’-GCCTCTTCTACGAGTCCATGCTCAA-3’
		reverse:	5’-CGTAGTCATTCCGCTTCACTTGCAG-3’
	hPRMT2	forward:	5’-GCAGGATGAAGAGTACTTCGGCAGC-3’
		reverse:	5'-CTTCTGCTGGTACACGGTGATGATG-3'
	hPRMT3	forward:	5'-CACATCACTCACTGCTACAAGGCTG-3'
		reverse:	5'-CACAGATGTCAGAACCTGCTCGTCA-3'
	hPRMT4	forward:	5'-CTTCTGCCGTGCAGTACTTCCAGTT-3'
		reverse:	5'-CTCCATGTAGAGCTGTTCATCCGTG-3'
	hPRMT5	forward:	5'- CACATCCACACTGGCCATCACTCTT-3'
		reverse:	5'- CCAGATTGTCCATCAGTGGCTGAAG-3'
	hPRMT6	forward:	5'-CAGTGGAGACTGTAGAGTTGCCGGA-3'
		reverse:	5'-GTCCACACCATAGTGCTGCTTCACC-3'
	hPRMT7	forward:	5'-TGGCTGGAGGAGGATGAACACTATG-3'
		reverse:	5'-TAGCTTGTTCCACGACCACATCCTC-3'
	hPRMT8	forward:	5'-CCGGAACTCCATGTACCACAACAAG-3'
		reverse:	5'- ACGATGTCCACTAGAGGCTCCTTCA-3'
	hPRMT9	forward:	5'-GAAGCCTTCGGACACATCTAACACG-3'
		reverse:	5'- CGTACAGGACCTTGCAGACTACTGG-3'
	sPRMT5	forward:	5'-TTCCTCTGCATGCCTGTCTTCCAC-3'
		reverse:	5'-AGTTGTGCCACCACATCCACGTCT-3'
	sPRMT6	forward:	5'-GAAGAGGAAGATGGCGGAGAGCAG-3'
		reverse:	5'-CACAGAGCTCAGCATAGACTCGTG-3'
	sPRMT7	forward:	5'-ATCCTGATCACAGAGCTGTTCGAC-3'
		reverse:	5'-AAGTCCACACTGAACATCGGCAGC-3'
	sPRMT9	forward:	5'-CGAGCTGGTGTCTCAGTCCTTG-3'
		reverse:	5'-TGCAGATCACTTCGTCATCAGG-3'
